# Non-Alcoholic Fatty Liver Disease (NAFLD) Pathogenesis and Natural Products for Prevention and Treatment

**DOI:** 10.3390/ijms232415489

**Published:** 2022-12-07

**Authors:** Xiangyu Guo, Xunzhe Yin, Zuojia Liu, Jin Wang

**Affiliations:** 1 State Key Laboratory of Electroanalytical Chemistry, Changchun Institute of Applied Chemistry, Chinese Academy of Sciences, Changchun 130022, China; 2 School of Clinical Medicine, Changchun University of Chinese Medicine, Changchun 130021, China; 3 Department of Chemistry and Physics, Stony Brook University, Stony Brook, NY 11794-3400, USA

**Keywords:** NAFLD, pathogenesis, natural products, plants, lipid accumulation, oxidative stress, endoplasmic reticulum stress, lipotoxicity

## Abstract

Non-alcoholic fatty liver disease (NAFLD) is the most prevalent chronic liver disease, affecting approximately one-quarter of the global population, and has become a world public health issue. NAFLD is a clinicopathological syndrome characterized by hepatic steatosis, excluding ethanol and other definite liver damage factors. Recent studies have shown that the development of NAFLD is associated with lipid accumulation, oxidative stress, endoplasmic reticulum stress, and lipotoxicity. A range of natural products have been reported as regulators of NAFLD in vivo and in vitro. This paper reviews the pathogenesis of NAFLD and some natural products that have been shown to have therapeutic effects on NAFLD. Our work shows that natural products can be a potential therapeutic option for NAFLD.

## 1. Introduction

**Figure 1:**
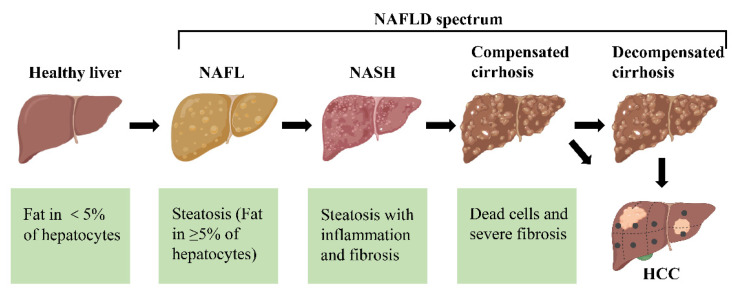
The different stages of non-alcoholic fatty liver disease (NAFLD). First, the healthy livers develop non-alcoholic fatty liver (NAFL) with hepatocellular steatosis as the main feature. If left untreated, NAFL may progress to a more severe form of non-alcoholic steatohepatitis (NASH), defined as inflammation and fibrosis in addition to hepatocellular steatosis. As the disease progresses, NASH may progress to cirrhosis and even to hepatocellular carcinoma (HCC).

Non-alcoholic fatty liver disease (NAFLD) has evolved from a relatively unknown disease to the most common cause of chronic liver disease worldwide. Currently, a consensus defines NAFLD as an umbrella term for a range of diseases in which steatosis is present in more than 5% of hepatocytes with metabolic risk factors (especially obesity and type 2 diabetes), excluding excessive alcohol consumption or other chronic liver disease [1,2]. NAFLD is divided into non-alcoholic fatty liver (NAFL) and non-alcoholic steatohepatitis (NASH) according to histological features (Figure 1) [3]. NAFL is defined as all cases characterized by steatosis, with or without mild lobular inflammation. In contrast, NASH is additionally characterized by the presence of hepatocellular damage (hepatocyte ballooning degeneration, diffuse lobular inflammation and fibrosis). Although simple steatosis is considered a “benign” disease, its association with liver fibrosis can lead to the development of cirrhosis and hepatocellular carcinoma (HCC) [4,5]. Therefore, NAFLD is considered an important factor in regulating mortality from liver-related diseases.

With the global increase in metabolic syndrome, obesity and diabetes, the prevalence of NAFLD has risen dramatically, affecting about a quarter of the world’s population [6]. Global epidemiological meta-analysis in 2016 showed that NAFLD is highly prevalent on all continents [7]. Possibly due to differences in overall caloric intake, physical activity, body fat distribution, socioeconomic status and genetic composition, the prevalence was highest in the Middle East and lowest in Africa, with approximately 31.79% (95% confidence interval (CI), 13.48–58.23) and 13.48% (95% CI, 5.69–28.69), respectively [7]. In this survey, metabolic comorbidities associated with NAFLD, including obesity 51.34% (95% CI, 41.38–61.20), type 2 diabetes 22.51% (95% CI, 17.92–27.89), hyperlipidemia 69.16% (95% CI, 49.91–83.46), hypertension 39.34% (95% CI, 33.15–45.88) and metabolic syndrome 42.54% (95% CI, 30.06–56.05), all showed strong correlations [7]. Obesity and insulin resistance (IR) lead to impaired lipid metabolism and chronic inflammation, which can lead to the progression of NAFLD to NASH and even to cirrhosis, HCC and death. Alarming data showed a global prevalence of 59.1% (95% CI, 47.6–69.7) of NASH among patients with biopsied NAFLD [7]. Patients with NAFLD are the fastest growing group of HCC patients requiring liver transplantation in the United States. A study analysed trends in the etiology of HCC from 2002–2012 and found the prevalence of NASH-related HCC increased from 8.3% in 2002 to 13.5% in 2012 [8]. As a result, NAFLD has become a world public health problem that cannot be ignored.

In clinical practice, patients with NAFLD show elevated triglycerides, elevated LDL and reduced HDL in biochemical tests [9]. The symptoms are usually associated with features of metabolic syndrome, such as obesity, dyslipidemia, type 2 diabetes and hypertension [2,7,10,11,12]. However, the pathogenesis of NAFLD is unknown, and this has become a hindrance to the treatment of NAFLD. Early studies suggest that IR and hepatic steatosis due to excess fatty acids are the “first-hit “, whereas hepatocytes eventually undergo damage, inflammation, fibrosis and other pathological changes due to oxidative stress and lipid peroxidation to form the “second-hit” [13]. Today, it is widely accepted that the “multiple-hit” theory is based on the “second-hit” theory, which includes various factors such as oxidative stress, endoplasmic reticulum (ER) stress and lipotoxicity [14]. This theory also provides a more accurate explanation for the pathogenesis of NAFLD.

Currently, there are no clinically approved drugs for NAFLD, and treatment is mainly through diet and exercise to change lifestyles [15]. However, patients with NAFLD often have difficulty maintaining an improved lifestyle. Therefore, it is of great practical importance to strengthen the research on the pathogenesis of NAFLD and to find safe and effective drugs for the prevention and treatment of NAFLD. With “NAFLD” and “Natural products” as key words, we searched PubMed database for relevant literature in the last ten years. It was found that the effects of natural products were usually evaluated in various signaling pathways related to lipid metabolism, oxidative stress, ER stress and lipotoxicity, and showed excellent therapeutic effects. In this article, we review the mechanisms associated with the pathogenesis of NAFLD and some natural small molecule compounds that have been shown to play a therapeutic role in NAFLD, as well as some natural compounds that may have therapeutic promise for NAFLD.

## 2. Pathogenesis

### 2.1. Lipid Accumulation

**Figure 2:**
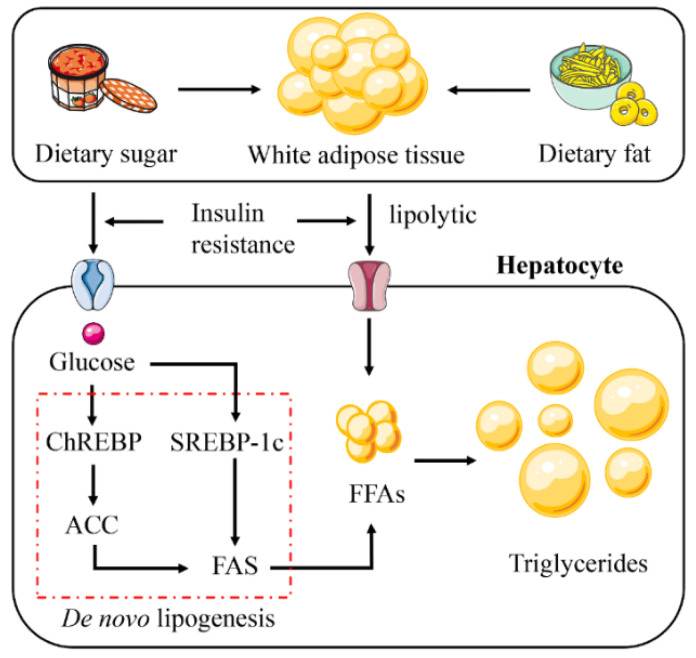
Abnormal lipid accumulation in NAFLD. The increase in hepatic lipid accumulation is due to the absorption of large amounts of free fatty acids (FFAs) synthesized triglycerides by the liver from white adipose tissue (WAT), high-fat and high-sugar foods, and de novo lipogenesis (DNL). Insulin resistance plays a vital role in this process. Insulin resistance promotes glucose absorption and enhances the lipolysis of WAT. This leads to the activation of the DNL pathway. Abbreviations: ChREBP, carbohydrate response element binding protein; SREBP-1c, sterol regulatory element binding protein 1c; ACC, acetyl-CoA carboxylase; FAS, fatty acid synthase.

When energy intake is higher than consumption, excess energy is stored in the form of lipids. In a disordered state, lipids are stored in other organs throughout the body [12,16,17,18]. NAFLD is a typical example of ectopic accumulation of lipids (Figure 2). Hepatic steatosis in NAFLD is triggered by excessive triglyceride (TG) synthesis in hepatocytes, with 60% of the substrate for this synthesis originating from white adipose tissue (WAT), 26% from de novo lipogenesis (DNL) and 15% from the consumption of a high-fat and/or high-sugar diet [19,20,21].

Insulin has an anti-lipolytic effect, mediates TG storage in adipose tissue, and promotes esterification and storage of fatty acids [22]. Therefore, insulin resistance (IR) becomes a key therapeutic factor in NAFLD. The fatty acid is mainly stored in the lipid droplets of WAT as TG [23]. Lipid droplets in cells have long been used as a relatively lazy lipid reservoir [24]. They act like a battery to store excess energy and release it when needed. In the IR state, the antilipolytic effect of insulin is diminished and WAT is broken down, leading to a large release of free fatty acids (FFAs) [25]. Then, excess FFAs are stored in the liver as TG, forming lipid ectopic deposits and causing NAFLD [26].

DNL is a key pathway that promotes lipid accumulation and is closely associated with IR [27]. DNL is modulated by sterol regulatory element-binding protein 1c (SREBP-1c) and carbohydrate response element-binding protein (ChREBP) [28,29]. IR activates SREBP-1c to promote DNL in hepatocytes [30,31]. Increased glucose concentration activates ChREBP to regulate the expression of acetyl-CoA carboxylase (ACC) and fatty acid synthase (FAS), thereby promoting DNL in hepatocytes [32,33,34].

With the obesity epidemic, we found that dietary factors are critical to the development of NAFLD [35,36,37]. A study suggested that a high-fat diet (HFD) alone led to obesity, IR and some degree of fatty liver with little inflammation and fibrosis, whereas a diet with added fructose increased the gene expressions for liver fibrosis, inflammation, ER stress and adipocyte apoptosis. [38]. In addition, animal models and human studies have shown that fructose has selective hepatic metabolism and triggers hepatic stress responses, including activation of c-Jun N-terminal kinase (JNK) and IR, which promotes fat accumulation in the liver, leading to increased lipogenesis and impaired fatty acid oxidation (FAO), triggering liver inflammation and liver fibrosis [39,40,41,42]. This suggests that fructose in the composition of the diet is an important risk factor for the development of NAFLD into NASH.

### 2.2. Oxidative Stress

**Figure 3:**
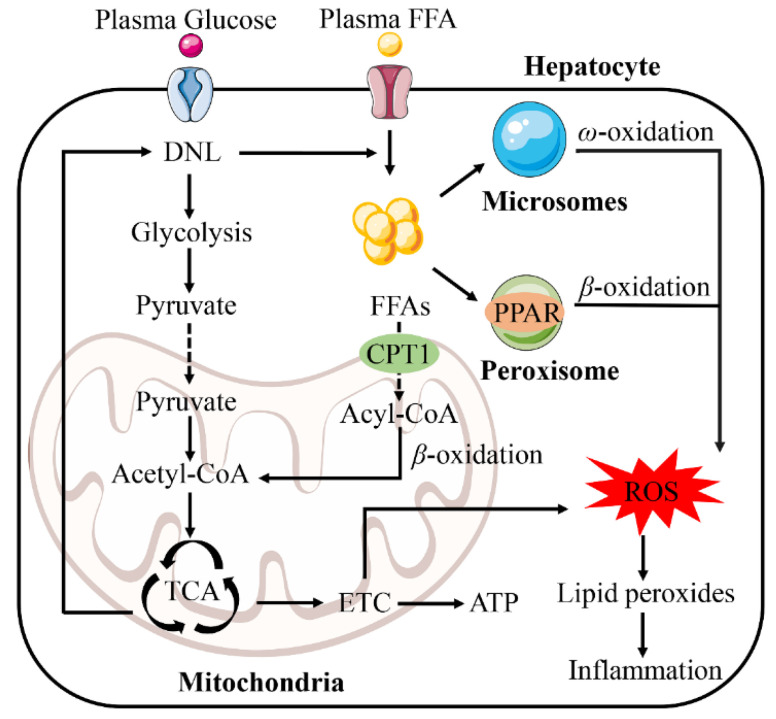
Fatty acid oxidation system in NAFLD. The fatty acid oxidation system consists of peroxisome, mitochondria and microsomes. Mitochondria play a vital role in fatty acid oxidation and energy supply. Glucose enhanced glycolysis and increased pyruvate content through the de novo lipogenesis (DNL) pathway. Pyruvate enters mitochondria and is converted into acetyl-CoA. Part of acetyl-CoA enters tricarboxylic acid cycle (TCA) and then synthesizes free fatty acids (FFAs) through the DNL pathway. The synthesized FFAs enter mitochondria together with the plasma FFAs through carnitine palmitoyltransferase 1 (CPT1) and are converted into acyl-CoA. Acyl-CoA is converted into acetyl-CoA by *β*-oxidation and enters the TCA to generate energy. The components of the mitochondrial respiratory chain are abnormally reduced by electrons and react with oxygen, producing a large number of reactive oxygen species (ROS). ROS further oxidize lipid deposition to form lipid peroxide, which leads to inflammatory reaction. Abbreviations: ETC, electron transport chain; PPAR, peroxisome proliferation-activated receptor.

Normally, DNL converts excess carbohydrates into fatty acids. Therefore, these fatty acids are esterified to form triglycerides (TG) that are stored in hepatocytes. In times of energy deficit, TG provides the body with energy through *β*-oxidation [43]. The increase of FFAs in the liver due to various causes leads to the damage of *β*-oxidation and mitochondrial dysfunction, resulting in inflammation, which leads to oxidative stress (Figure 3) [44]. Reactive oxygen species (ROS) are important mediators of the inflammatory response [45,46].

The peroxisome is the first enzyme of the fatty acid *β*-oxidation system. Peroxisome proliferation-activated receptor α (PPARα) regulates the activity of three interrelated hepatic fatty acid oxidation systems, namely the mitochondrial and peroxisomal *β*-oxidation, and microsomal ω-oxidation pathways [47]. Sustained activation of PPARα can alleviate NAFLD by enhancing FAO and reducing ROS levels [48,49,50]. However, many studies have found that excessive activation of PPARα enhances hepatic FAO and also leads to excessive combustion of hepatic energy, disproportionately increasing H_2_O_2_ and producing an inflammatory response [51,52,53].

NAFLD patients exhibit ultrastructural mitochondrial damage, reduced respiratory chain complex activity and impaired ATP synthesis [54]. Mitochondria play a very important role in FAO and energy supply, but a large number of ROS are also produced in this process, which is one of the main sources of ROS in cells [55]. ACC catalyzes DNL and regulates mitochondrial FAO [56]. DNL enhances glycolytic activity, resulting in a rise in pyruvate and acetyl-CoA. FFAs cross the inner mitochondrial membrane through the carnitine palmitoyltransferase 1 (CPT1) [57]. Impaired mitochondrial *β*-oxidation occurs when the transport of fatty acids to the mitochondria is reduced [58,59]. In the mitochondria, acyl-CoA is converted to acetyl-CoA by *β*-oxidation and then enters the tricarboxylic acid cycle (TCA) to provide energy. More specifically, the mitochondrial dysfunction is due to the damage of the electron transport chain (ETC). Components of the mitochondrial respiratory chain are over-reduced by electrons, which then react abnormally with oxygen, leading to increased ROS [60]. In addition, ROS oxidizes fatty deposits to release lipid peroxides that damage hepatocytes. In hepatocytes, ROS and lipid peroxides further damage the respiratory chain, directly or indirectly causing oxidative damage to the mitochondrial genome, which also leads to the production of more ROS, thus creating a vicious cycle [61,62].

### 2.3. Endoplasmic Reticulum (ER) Stress

**Figure 4:**
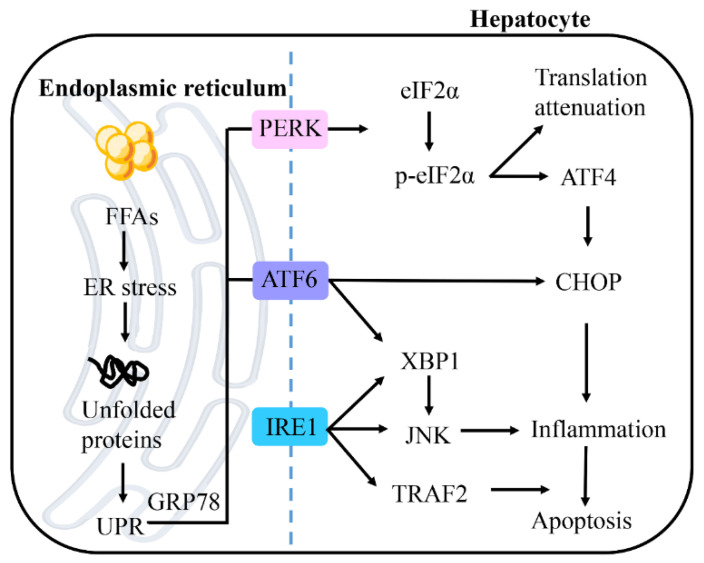
Endoplasmic reticulum (ER) stress in NAFLD. With the increase of lipid accumulation, ER stress leads to a large number of unfolded proteins, thus triggering unfolded protein response (UPR). UPR is mediated by protein kinase RNA-like ER kinase (PERK), inositol-requiring enzyme 1 (IRE1), and activating transcription factor 6 (ATF6). PERK-mediated phosphorylation of eukaryotic initiation factor 2α (eIF2α) leads to the transient weakening of translation, but activation of transcription factor 4 (ATF4) induces the gene expression of CCAAT-enhancer-binding protein homologous protein (CHOP). ATF6 can also activate CHOP to induce apoptosis. ATF6 and IRE1 promote the expression of X-box binding protein-1 (XBP1), and mediate inflammation through the c-Jun N-terminal kinase (JNK) signaling pathway. IRE1 can also directly promote the activation of JNK and activate tumor necrosis factor (TNF) receptor-related factor 2 (TRAF2), thus promoting cell apoptosis. Abbreviations: GRP78, glucose-regulated protein 78.

ER stress is a protective response that restores protein homeostasis by activating the unfolded protein response (UPR) [63]. However, when activation of the UPR fails to promote cell survival, cells are activated by the proapoptotic ER stress pathway, which ultimately leads to cell death (Figure 4) [64]. The ER membrane consists of a small amount of cholesterol and complex sphingolipids [65]. This loose packing of ER membrane lipids facilitates the synthesis of new lipids and the transport of proteins. Lipogenesis is the main metabolic pathway affected by ER stress [66]. Recent data suggest that ER is present in both the development of hepatic steatosis and the progression of NASH [67]. Disrupted ER homeostasis has been reported to be found in the liver of NAFLD patients [68]. This result suggests that ER stress is closely associated with NAFLD.

UPR is mediated by three typical ER-resident stress sensors, protein kinase RNA-like ER kinase (PERK), inositol-requiring enzyme 1 (IRE1), and activating transcription factor 6 (ATF6) [69]. In normal conditions, these molecules bind to glucose-regulated protein 78 (GRP78) and keep it in an inactive state. Under ER stress conditions, all these pathways can be activated after separation from GRP78, which influences different downstream events [70].

The three proximal UPR sensors PERK, IRE1 and ATF6 all regulate lipid storage in the liver [67]. The IRE1-XBP1 and PERK-peIF2α pathways upregulate the adipogenic gene program, whereas the interaction between ATF6, sterol regulatory element-binding protein 2 (SREBP2) and histone deacetylase 1 (HDAC1) can limit adipogenesis. In the absence of resolution of ER stress, hepatic steatosis may be promoted through the upregulation of lipid input pathways and the downregulation of lipid output pathways. It has been reported that ER stress promotes the activation of the adipogenic transcription factor SREBP-1c, thereby promoting adipogenesis [71,72]. Significant insulin resistance and steatosis in obese rodents are paradoxically associated with adipogenic activation in the liver precisely because SREBP-1c and adipogenic activation in fatty liver are secondary to ER stress [28].

ER stress promotes apoptosis through three sensor dimers and autophosphorylation. [73]. PERK-mediated phosphorylation of eukaryotic initiation factor 2α (eIF2α) leads to transient attenuation of translation but activates transcription factor 4 (ATF4) for selective translation. ATF4 acts as a transcription factor and induces the gene expression of CCAAT-enhancer-binding protein homologous protein (CHOP), which is associated with apoptosis [74]. CHOP is also a substrate of ATF6. In addition, ATF6 upregulates the expression of X-box binding protein-1 (XBP1), which mediates inflammatory responses through the JNK signaling pathway. IRE1 promotes the activation of tumor necrosis factor (TNF) receptor-associated factor 2 (TRAF2) and JNK, thereby promoting apoptosis. Several studies have confirmed that the IRE1 pathway can activate JNK through its kinase structural domain, leading to the increased expressions of proinflammatory mediators [75,76,77]. Activation of IRE1 leads to splicing of XBP1. XBP1 is a key transcription factor that regulates genes encoding adaptive UPR. These suggest that ER stress leads to the progression of NAFLD to a more severe form of NASH.

### 2.4. Lipotoxicity

**Figure 5:**
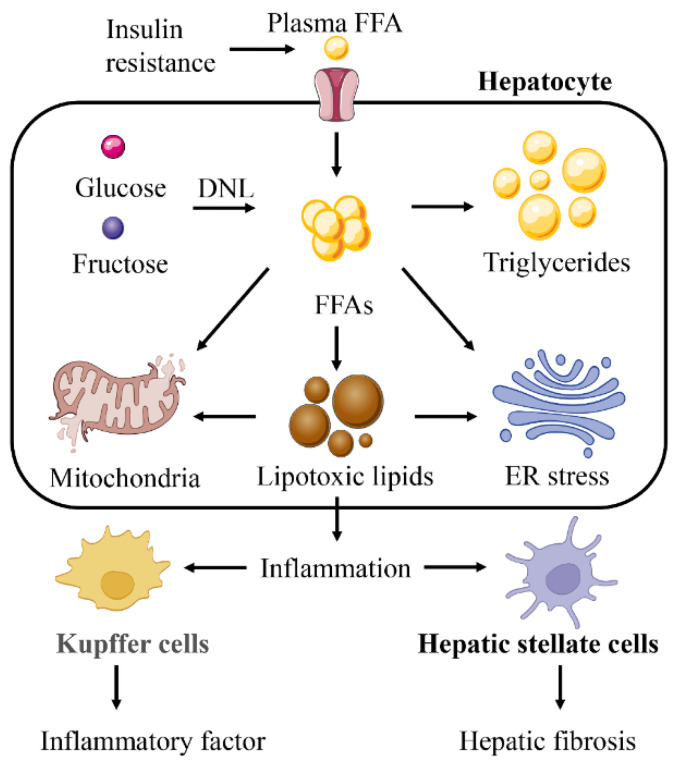
Lipotoxicity is the core influencing factor for NAFLD to develop into a more serious situation. Lipotoxicity will aggravate mitochondrial dysfunction and endoplasmic reticulum (ER) stress caused by free fatty acids (FFAs). Lipotoxicity can also induce Kupffer cells and hepatic stellate cells to produce a wider range of inflammatory reactions and liver fibrosis.

Lipotoxicity is the toxic effect of sustained high concentrations of lipids and metabolites deposited excessively in nonadipose tissue, causing damage to that tissue [78]. When lipotoxic substances in hepatocytes are consistently elevated beyond the hepatocyte’s ability to transport them, hepatocyte damage is exacerbated and the disease progresses to a more severe situation. In NAFLD, IR leads to a significant increase in plasma FFAs, and FAO overload in hepatocytes leads to mitochondrial damage, generating large amounts of ROS and causing ER stress, oxidative stress, and inflammatory responses (Figure 5). A range of effects of lipotoxicity from free fatty acids (FFAs) play an important role in the development of NASH and drive further progression of the disease.

Not all lipids are lipotoxic. For example, TG and FFA containing unsaturated double bonds have protective effects against lipotoxic substance-induced liver injury [79]. In this regard, a study on a mouse model of NASH found that fatty triglyceride lipase (ATGL) deficiency inhibited TG catabolism and reduced the release of FFAs, thus providing protection against liver injury [80]. In addition, it has been observed that monounsaturated oleic acid (OA) can promote the development of hepatic steatosis but is less toxic than saturated FFAs, such as palmitic acid (PA) and stearic acid [81]. These lipids and their metabolites that can cause cellular damage are called lipotoxic substances, which include saturated PA, ceramides, bile acids, and free cholesterol. Ceramides have been reported to affect IR and inflammatory pathways in mouse models and in patients with NASH [78]. The structure of bile acids is hydrophobic and inherently toxic to cells. It has been demonstrated that bile acids disrupt cell membranes by dissolving phospholipids, cholesterol and fatty acids in lipid bilayers [82]. Free cholesterol can activate SREBP-2 to upregulate LDL receptors, thereby reducing the biotransformation of cholesterol to bile acids [83]. These lipotoxic substances may lead to apoptosis, inflammation, increased liver fibrosis, and the development of steatosis to NASH.

Lipotoxicity has different effects on different cells. The mechanism of lipotoxicity described earlier in this paper acts on hepatocytes, the major component of hepatic parenchymal cells. However nonparenchymal cells such as hepatic stellate cells (HSCs) and Kupffer cells (KCs) also play very important roles in the progression of NASH. HSCs are the main cell population involved in hepatic fibrogenesis and are the main cause of NASH progression. It has been found that activation of TLR4 by lipotoxic substances promotes inflammatory and fibrotic signaling in HSCs [84]. KCs cells regulate the inflammatory response of the hepatic microenvironment and participate in the development of liver disease by secreting proinflammatory cytokines. Elevated concentrations of the oxidized LDL in NASH patients produce inflammation by KCs cells [85].

## 3. Potential Natural Ingredients for the Treatment of NAFLD

**Figure 6:**
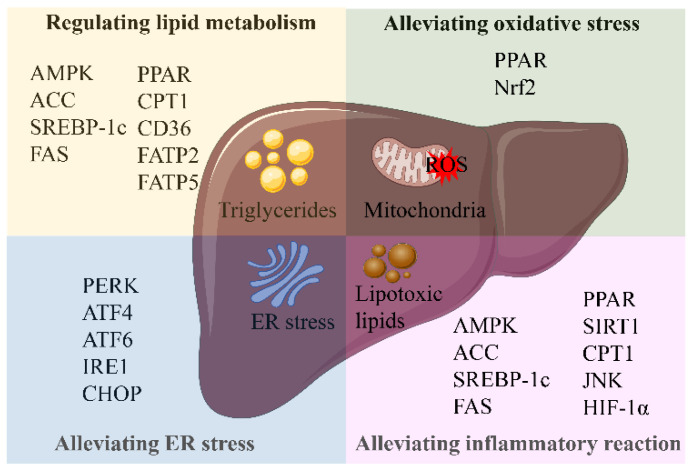
Related targets of natural products in the treatment of NAFLD. Abbreviations: AMPK, (AMP)-activated protein kinase; ACC, acetyl-CoA carboxylase; SREBP-1c, sterol regulatory element-binding protein 1c; FAS, fatty acid synthase; PPAR, peroxisome proliferation-activated receptor; CPT1, carnitine palmitoyltransferase 1; CD36, cluster of differentiation 36; FATP2, fatty acid transport proteins 2; FATP5, fatty acid transport proteins 5; Nrf2, nuclear factor erythroid-derived 2-like 2; PERK, protein kinase RNA-like ER kinase; ATF4, activating transcription factor 4; ATF6, activating transcription factor 6; IRE1, inositol-requiring enzyme 1; CHOP, CCAAT-enhancer-binding protein homologous protein; SIRT1, silent information regulator 1; JNK, c-Jun N-terminal kinase; HIF-1α, hypoxia-inducible factor 1α.

**Figure 7:**
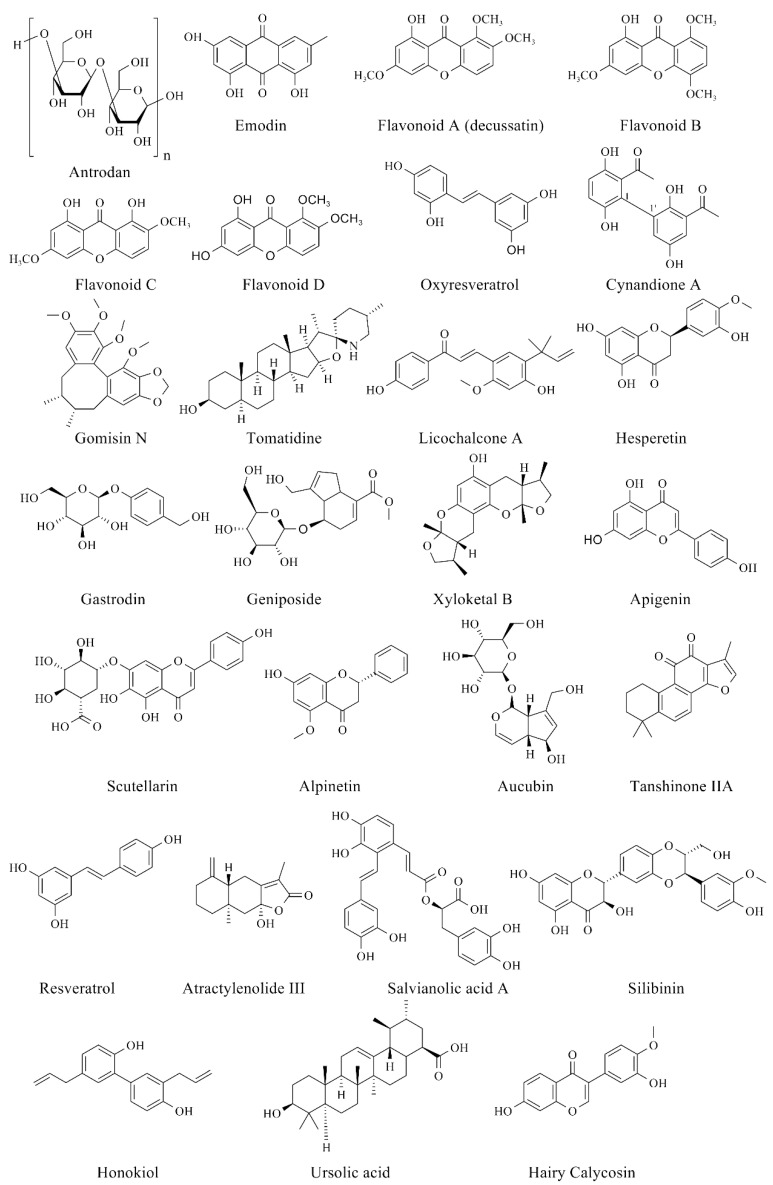
Chemical structures of bioactive compounds for relieving NAFLD.

Based on the fact that there are no Food and Drug Administration (FDA)-approved drugs for the treatment of NAFLD, we summarize the natural products that have the effect of alleviating NAFLD. The exploration of natural products may be a broad direction for the treatment of NAFLD. Here, we classify the relevant natural products according to the pathogenesis of NAFLD, which can be classified according to their different functions, including regulation of lipid metabolism, improvement of oxidative stress, improvement of ER stress, and alleviation of inflammation (Figure 6). These natural products are divided into bioactive species (Table 1) and bioactive compounds (Table 2). The chemical structures of the bioactive compounds for relieving NAFLD are shown in Figure 7.

### 3.1. Regulating Lipid Metabolism

Excessive calorie intake will activate the DNL, resulting in increased lipid accumulation. Adenosine monophosphate (AMP)-activated protein kinase (AMPK), the main cellular energy sensor, has been implicated as a key regulator of hepatic lipid and glucose metabolism [86]. Recently, emerging evidence indicates that many plant-derived natural products are capable of ameliorating lipid metabolism by targeting AMPK.

Antrodan is a type of *β*-glucan extracted and purified from *Antrodia cinnamomea* T. T. Chang & W. N. Chou [49], a precious edible fungus native to Taiwan [87]. Antrodan has been reported to reduce the plasma levels of malondialdehyde, total cholesterol, triglycerides, GOT, GPT, uric acid, glucose and insulin, and to upregulate the leptin and adiponectin in a high-fat and high-fructose diet mouse model. Protein expression levels were measured after the administration of the drug, and the results showed that Antrodan improved the effects of NAFLD mice by activating the AMPK pathway [49]. Emodin extracted from Radix Polygoni Multiflori (*Fallopia multiflora* (Thunb.) Harald) significantly reduced the contents of TG, TC and FFAs in zebrafish with NAFLD. This study shows that emodin alleviates NAFLD by modulating the AMPK signaling pathway, increasing IR sensitivity and FAO [88]. Flavonoids A-D with similar structures extracted from *Lomatogonium rotatum* (L.) Fries ex Nym. (belonging to Gentianaceae) have the effects of reducing blood lipids and inhibiting obesity. Four types of flavonoids can effectively improve serum TC and TG levels. Among them, flavonoid C can also improve HDL and LDL levels at the same time, and it has the strongest ability to improve serum lipid parameters. Only the flavonoid B cannot improve serum HDL levels. In addition, it was found that the four flavonoids stimulated AMPK in different degrees and decreased the expression level of FAS protein [89]. Another potential treatment for NAFLD is oxyresveratrol, a naturally occurring polyhydroxylated stilbene that is abundant in mulberry wood (*Morus alba* L.). Gene- and protein-level assays showed that it was able to upregulate the expression level of p-AMPK and downregulate the expression level of SREBP-1c, suggesting that the regulation of this extract is mediated by the AMPK/SREBP-1c pathway [90]. Cynandione A, isolated from ethyl acetate extract of *Cynanchum wilfordii* (Maxim.) Hemsl., is a bioactive phytochemical that has been found to be beneficial for the treatment of several diseases. It has been reported that it can also activate AMPK and inhibit the expression of SREBP-1c protein, which can improve NAFLD by inhibiting hepatic DNL [91]. Gomisin N derived from *Schisandra chinensis* (Turcz.) Baill. can also play a role in improving lipid metabolism by activating AMPK [92]. Moreover, activation of AMPK signaling was observed in both tomatidine [93] and licochalcone A [94] during these studies, and all of them have a well-regulated effect on lipid metabolism. These studies revealed that AMPK has a very important function in the regulation of lipid metabolism.

*Poria cocos* (Schw.) Wolf is an edible, pharmaceutical mushroom with remarkable biological properties, including anti-tumor, anti-inflammation, anti-oxidation, anti-ageing, and anti-diabetic effects. It can significantly activate AMPK and autophagy-related protein expression and inhibit ER stress-related protein expression when acting on HepG2 cells and HFD-fed obese mouse, suggesting that it can alleviate liver steatosis through AMPK-activated autophagy [95]. *Curcuma longa* L. is a flowering plant of the ginger family (belonging to Zingiberaceae). *C. longa* restricted the expression of fatty acid transport-related, including cluster of differentiation 36 (CD36) and fatty acid transport proteins (FATP2 and FATP5), thus reducing the expression levels of SREBP-1c, ACC, FAS, PPARα and CPT1, which played a role in reducing lipid accumulation. In addition, the activation of the AMPK signal was also observed in this study [96]. *Citrus unshiu* Marc. peel extracts contain compounds that potentially improve dyslipidemia. The study demonstrates that Citrus peel inhibits fatty liver development and hepatotoxicity in HFD-induced NAFLD and also prevents abnormal lipid accumulation in vivo by regulating AMPK activation and the alleviation of mTORC1-ER stress [97]. This experimental evidence reveals the potential protective mechanism of AMPK in the lipid metabolism of NAFLD, thus paving the way for developing new strategies to prevent complications of NAFLD.

### 3.2. Alleviating Oxidative Stress

The excessive intake of a high-calorie diet gradually leads to accumulation of malonyl coenzyme A, which inhibits fatty acid *β*-oxidation in hepatocytes. Nuclear factor erythroid-derived 2-like 2 (Nrf2) and PPAR play the role of antioxidation and regulating lipid metabolism in NAFLD, and they are interrelated [98]. Some natural products can target Nrf2 and PPAR signals and alleviate the oxidative stress caused by the lack of antioxidant capacity in the treatment of NAFLD.

Hesperetin, a citrus flavonoid belonging to the flavanone class, is abundant in oranges, lemons and grape juice consumed in the Eastern and Western daily diet. Hesperetin was able to increase antioxidant levels and reduce ROS levels and hepatotoxicity. This study proposes that hesperetin alleviates hepatic steatosis, oxidative stress, inflammatory cell infiltration and fibrosis by triggering the Nrf2 pathway [99]. Gastrodin is a water-soluble natural compound extracted from the root of *Gastrodia elata* Blume. Gastrodin significantly decreased ROS and reduced the mRNA levels of proinflammatory cytokines both in vivo and in vitro. In addition, this study also found an activating effect of gastrodin on NrF2 [100]. In mice with diet-induced NASH, yellow loosestrife (*Lysimachia vulgaris* var. *davurica* (Ledeb.) R. Knuth) exerts antioxidant and anti-inflammatory effects by activating NrF2 signaling [101]. Having the same function as the natural products mentioned above, geniposide also regulates antioxidant capacity by modulating NrF2. The study proved the protective effect of geniposide on lipid accumulation via enhancing the ability of antioxidative stress and anti-inflammation [102].

Xyloketal B is a unique condensed ketone compound isolated from the mangrove fungus *Xylaria* sp. in the South China Sea. The treatment of NAFLD mainly enhances FAO by upregulating PPARα [103]. The watery extract of chicory (*Cichorium intybus* L.) seed can improve lipid accumulation by upregulating PPAR protein expression levels. The extract increased expression of genes related to antioxidant pathways that protect the liver from ROS formed in the FAO pathway [104]. *Crataegus azarolus* var. *aronia* L. prevented the increase in serum and hepatic lipids and reduced hepatic levels of ROS. The study also noted that *Crataegus aronia* could reverse HFD-induced hepatic steatosis by the activation of AMPK, which leads to subsequent inhibition of SREBP1/2 and activation of PPARα [105].

The food-derived compound apigenin regulation of PPARγ target genes is dependent on the activation of Nrf2. This study also suggests that apigenin may bind to Nrf2 to co-regulate lipid metabolism and oxidative stress [106]. Scutellarin is a flavonoid glycoside having antioxidative stress activity. Scutellarin reduces lipid content and enhances antioxidant capacity in in vitro and in vivo models, possibly related to the activation of PPARγ and Nrf2 [107]. Alpinetin is a novel plant flavonoid isolated from *Alpinia katsumadai* Hayata, which is a traditional Chinese medicine. The same effect as scutellarin, the anti-lipid accumulation effect of alpinetin is through activation of PPAR and Nrf2 signals and reduction in the expression of hepatic lipogenic proteins [108].

### 3.3. Alleviating Endoplasmic Reticulum (ER) Stress

The ER is a major intracellular organelle involved in lipid metabolism in hepatocytes and plays a crucial role in lipid accumulation in NAFLD. ER stress is closely associated with hepatic oxidative stress and leads to severe liver injury through mechanisms such as activation of cell death signals, dysregulation of autophagic fluxes and hepatic inflammation.

Coffee (*Coffea arabica* L.) is the most consumed beverage worldwide. Coffee improves ER stress and mitochondrial functional impairment, ensuring proper protein folding and degradation in the liver [109]. *Amomum villosum* var. *xanthioides* (Wall. ex Baker) T.L.Wu & S.J.Chen is a traditional Chinese herb. It also alleviates NAFLD caused by ER stress by increasing antioxidant capacity [110].

The aqueous extract of *Eucommia ulmoides* Oliver leaves restores abnormal lipid metabolism in HFD-fed mice. The improvement of NAFLD is achieved by inhibiting ER stress, enhancing lysosomal function and autophagy flux [111]. The two active components of *Eucommia ulmoides* Oliver, aucubin and geniposide, can inhibit ER stress. Its active components can enhance lysosomal activity and reduce ER stress and liver dyslipidemia in in vivo and in vitro models [112].

*Ixeris dentata* (Thunb.) Nakai is a traditional herb for treating hepatitis, indigestion and diabetes. *I. dentata* can significantly reduce ER stress of hepatocytes induced by palmitic acid. Specifically, it can inhibit the expression of PERK, eIF2α phosphate and CHOP, and it can reduce the accumulation of triglyceride and cholesterol in hepatocytes [113]. Tanshinone IIA is one of the effective components of the traditional Chinese medicine *Salvia miltiorrhiza* Bunge. Tanshinone IIA not only inhibited the expression of PERK, eIF2α phosphate and CHOP, but also alleviated ER stress-induced apoptosis in hepatocytes [114]. In addition, *Vigna nakashimae* (Ohwi) Ohwi and H.Ohashi extract has been shown to alleviate ER stress and hepatocyte apoptosis. Specifically, it can reduce hepatic ACC, ATF4 and caspase-3 induced by HFD [115].

### 3.4. Alleviating Inflammatory Reaction

Lipid toxicity is an inflammatory reaction caused by abnormal lipid metabolism, which leads to cell dysfunction and cell death. The effect of natural products on improving lipid toxicity is related to regulating lipid metabolism, improving antioxidant capacity and inhibiting hepatocyte apoptosis.

Resveratrol is a natural polyphenol compound found in grapes and red wine. Treatment with resveratrol has been reported to improve lipid metabolism and reduce pro-inflammatory features in the liver of nonalcoholic fatty liver and HFD-induced mouse [116]. *Cynanchum atratum* Bunge is a kind of herbal medicine that has the functions of detoxification, diuresis and fever reduction. *C. atratum* regulates lipid metabolism and inhibits liver inflammatory factors by activating AMPK [117]. *Lycopus lucidus* Turcz. ex Benth. is a perennial plant belonging to the Lamiaceae family. It reduced lipid accumulation and regulates fatty acid oxidation by activating AMPK and PPAR signaling to alleviate inflammatory responses [118]. Atractylenolide III is the major bioactive component found in *Atractylodes macrocephala* Koidz. The study showed that Atractylenolide III ameliorated liver injury and liver lipid accumulation in HFD-induced NAFLD mouse models by activating the AMPK/SIRT1 pathway [119]. Salvianolic acid A is a natural polyphenol compound extracted from *Salvia miltiorrhiza* Bunge (known as Danshen in China). Similar to Atractylenolide III, it alleviates the inflammatory response caused by lipotoxicity through the AMPK/SIRT1 pathway [120].

Silibinin is a flavonolignan isolated from the fruit and seeds of Milk thistle (*Silybum marianum* (L.) Gaertn.). Silibinin prevents NASH by modulating the JNK pathway. It not only promoted *β*-oxidation in liver and reduced lipid accumulation, but also regulated antioxidant enzyme activity and oxidase activity to reduce oxidative stress [121]. Honokiol extracted from *Magnolia officinalis* Rehd. et Wils. also improved NAFLD lipid accumulation and oxidative stress by activating the JNK pathway [122]. Ursolic acid is a natural pentacyclic triterpene carboxylic acid that can improve NASH by inhibiting hypoxia-inducible factor 1α (HIF-1α) signal [123]. Chinese herbal monomer hairy calycosin is a flavonoid extracted from Radix astragali (*Astragalus membranaceus* (Fisch.) Bge. var. *mongholicus* (Bge.) Hsiao). Hairy calycosin can effectively control the lipid peroxidation in liver tissues of rats with NAFLD and improve the steatosis and inflammation of liver tissue, inhibiting apoptosis of hepatocytes [124].

**Table 1:** Potential bioactive species for the treatment of NAFLD.

Biological Function of NAFLD	N	Bioactive Species.	Application Model	Ref.
Regulating lipid metabolism	(1)	*Poria cocos*	HFD-fed mouse, OA plus PA stimulated HepG2 cells	[95]
	(2)	*Curcuma longa*	HFD-fed mouse, OA plus PA stimulated HepG2 cells	[96]
	(3)	*Citrus unshiu* peel	HFD-fed rat, PA stimulated AML 12 cells	[97]
Alleviating oxidative stress	(4)	*Lysimachia vulgaris* var. *davurica*	Methionine-choline deficiency (MCD)-fed mouse	[101]
	(5)	*Cichorium intybus*	Streptozotocin-induced rat, Streptozotocin and niacinamide-induced rat, OA stimulated HepG2 cells	[104]
	(6)	*Crataegus azarolus* var. *aronia*	HFD-fed rat	[105]
Alleviating endoplasmic reticulum stress	(7)	*Coffea arabica*	HFD-fed rat	[109]
	(8)	*Amomum villosum* var. *xanthioides*	Tunicamycin stimulated mouse and Huh7 cells	[110]
	(9)	*Eucommia ulmoides* leave	HFD-fed rat	[111]
	(10)	*Ixeris dentata*	PA stimulated HepG2 cells	[113]
	(11)	*Vigna nakashimae*	HFD-fed mouse	[115]
Alleviating inflammatory reaction	(12)	*Cynanchum atratum*	high-fat and high-fructose diet (HFHFD)-fed mouse	[117]
	(13)	*Lycopus lucidus*	HFD-fed mouse, OA plus PA stimulated HepG2 cells	[118]

**Table 2:** Potential bioactive compounds for the treatment of NAFLD.

Biological Function of NAFLD	N	Bioactive Compounds	Application Model	Ref.
Regulating lipid metabolism	(1)	Antrodan	High-fat diet (HFD)-fed mouse	[49]
	(2)	Emodin	Egg yolk powder-fed Zebrafish	[88]
	(3)	Four flavonoids extracted from *Lomatogonium rotatum*	High fructose-fed rats	[89]
	(4)	Oxyresveratrol	HFD-fed mouse, liver X receptor α (LXRα) stimulated HepG2, Hep3B and Huh-7 cells	[90]
	(5)	Cynandione A	LXRα stimulated HepG2 cells	[91]
	(6)	Gomisin N	HFD-fed mouse, LXRα or palmitic acid (PA) stimulated HepG2 cells	[92]
	(7)	Tomatidine	HFD-fed mouse, Oleic acid (OA) stimulated FL83B	[93]
	(8)	Licochalcone A	HFD-fed mouse, OA stimulated HepG2 cells	[94]
Alleviating oxidative stress	(9)	Hesperetin	HFD-fed rat, OA stimulated HepG2 cells	[99]
	(10)	Gastrodin	HFD-fed mouse, high-fat and high-carbohydrate diet (HFHC)-fed rat, OA stimulated HL-7702 cells	[100]
	(11)	Geniposide	tyloxapol-induced mouse, OA plus PA stimulated HepG2 cells	[102]
	(12)	Xyloketal B	HFD-fed mouse, MCD-fed mouse, OA plus PA stimulated HepG2 cells	[103]
	(13)	Apigenin	HFD-fed mouse, OA plus PA stimulated mouse Hepa1-6 cell	[106]
	(14)	Scutellarin	HFD-fed mouse, OA stimulated HepG2 cells	[107]
	(15)	Alpinetin	HFD-fed mouse, high concnetration of fructose stimulated HL-7702 cell	[108]
Alleviating endoplasmic reticulum stress	(16)	Aucubin	HFD-fed rat, PA stimulated HepG2 cells	[112]
	(17)	Geniposide	HFD-fed rat, PA stimulated HepG2 cells	[112]
	(18)	Tanshinone IIA	PA stimulated HepG2 cells	[114]
Alleviating inflammatory reaction	(19)	Resveratrol	HFD-fed mouse	[116]
	(20)	Atractylenolide III	HFD-fed mouse, OA plus PA stimulated HepG2 cells	[119]
	(21)	Salvianolic acid A	HFHC-fed mouse, PA stimulated HepG2 cells	[120]
	(22)	Silibinin	MCD-fed mouse, OA plus PA stimulated mouse NCTC-1469 cells	[121]
	(23)	Honokiol	MCD-fed mouse, OA plus PA stimulated mouse NCTC-1469 cells	[122]
	(24)	Ursolic acid	HFD-fed mouse	[123]
	(25)	Hairy calycosin	HFD-fed rat	[124]

## 4. Conclusions

This paper provides a review of the current state of research on the pathogenesis of NAFLD and summarizes the natural products in the recent literature that have modulating effects on in vitro and in vivo models of NAFLD. The development of NAFLD is mostly associated with lipid accumulation, oxidative stress, ER stress and lipotoxicity. It is interesting to note that these active compounds act in a multi-targeted manner, such as regulating the levels of AMPK, PPAR, SREBP-1c, FAS, ACC, SIRT1, Nrf2, JNK and other proteins to improve NAFLD. These natural products may provide a new way for the research and discovery of new drugs for the treatment of NAFLD.

At the same time, we also face some issues. The existing natural product research lacks consistent standards and norms, resulting in immature evaluation systems and unclear potential mechanisms. In addition, the extraction technology of effective components of many natural products is not yet mature. This leads to a significant decrease in bioavailability. Therefore, more work needs to be done to apply natural products to the treatment of NAFLD.
